# Exercise cardiovascular magnetic resonance: feasibility and development of biventricular function and great vessel flow assessment, during continuous exercise accelerated by Compressed SENSE: preliminary results in healthy volunteers

**DOI:** 10.1007/s10554-020-02044-8

**Published:** 2020-10-04

**Authors:** Thomas P. Craven, Nicholas Jex, Pei G. Chew, David M. Higgins, Malenka M. Bissell, Louise A. E. Brown, Christopher E. D. Saunderson, Arka Das, Amrit Chowdhary, Erica Dall’Armellina, Eylem Levelt, Peter P. Swoboda, Sven Plein, John P. Greenwood

**Affiliations:** 1 Multidisciplinary Cardiovascular Research Centre & The Division of Biomedical Imaging, Leeds Institute of Cardiovascular and Metabolic Medicine, University of Leeds, Leeds, LS2 9JT UK; 2 Philips, Guildford, England, UK

**Keywords:** Exercise cardiovascular magnetic resonance, Exercise, Flow, Ventricular function

## Abstract

**Purpose:**

Exercise cardiovascular magnetic resonance (Ex-CMR) typically requires complex post-processing or transient exercise cessation, decreasing clinical utility. We aimed to demonstrate the feasibility of assessing biventricular volumes and great vessel flow during continuous in-scanner Ex-CMR, using vendor provided Compressed SENSE (C-SENSE) sequences and commercial analysis software (Cvi42).

**Methods:**

12 healthy volunteers (8-male, age: 35 ± 9 years) underwent continuous supine cycle ergometer (Lode-BV) Ex-CMR (1.5T Philips, Ingenia). Free-breathing, respiratory navigated C-SENSE short-axis cines and aortic/pulmonary phase contrast magnetic resonance (PCMR) sequences were validated against clinical sequences at rest and used during low and moderate intensity Ex-CMR. Optimal PCMR C-SENSE acceleration, C-SENSE-3 (CS3) vs C-SENSE-6 (CS6), was further investigated by image quality scoring. Intra-and inter-operator reproducibility of biventricular and flow indices was performed.

**Results:**

All CS3 PCMR image quality scores were superior (p < 0.05) to CS6 sequences, except pulmonary PCMR at moderate exercise. Resting stroke volumes from clinical PCMR sequences correlated stronger with CS3 than CS6 sequences. Resting biventricular volumes from CS3 and clinical sequences correlated very strongly (r > 0.93). During Ex-CMR, biventricular end-diastolic volumes (EDV) remained unchanged, except right-ventricular EDV decreasing at moderate exercise. Biventricular ejection-fractions increased at each stage. Exercise biventricular cine and PCMR stroke volumes correlated very strongly (r ≥ 0.9), demonstrating internal validity. Intra-observer reproducibility was excellent, co-efficient of variance (COV) < 10%. Inter-observer reproducibility was excellent, except for resting right-ventricular, and exercise bi-ventricular end-systolic volumes which were good (COV 10–20%).

**Conclusion:**

Biventricular function, aortic and pulmonary flow assessment during continuous Ex-CMR using CS3 sequences is feasible, reproducible and analysable using commercially available software.

## Introduction

Stress cardiac imaging is an important tool in assessing valvular [1] and congenital heart disease [2] and has significantly improved the diagnostic accuracy for coronary artery disease (CAD) detection compared to exercise ECG [3, 4]. Cardiovascular magnetic resonance (CMR) has well established benefits over alternative imaging modalities and as such is the reference standard for bi-ventricular volume and functional assessment [5]. Pharmacological stress CMR is well established clinically, demonstrating superiority over myocardial perfusion scintigraphy by single photon emission computed tomography (MPS-SPECT) in the diagnosis [6, 7] and prognostication of CAD [8]. However, physical exercise allows a more detailed assessment of symptoms, functional state and haemodynamic response and has fewer adverse events compared to pharmacological stress [9, 10]. As such, current guidelines advise physical exercise as the preferred method for stress imaging when feasible [11, 12]. Exercise CMR (Ex-CMR) combines the superior image quality of CMR with the preferred method of stress by physiological exercise. Despite research development over the past 3 decades, Ex-CMR is not widely utilised clinically. Treadmill Ex-CMR has demonstrated clinical utility and superiority over MPS-SPECT, in the detection of ischaemia in CAD [13]. However, heart rate reductions during transfer to the MR-scanner limit its clinical utility beyond CAD assessment and make assessment at multiple exercise intensities logistically difficult. In-scanner Ex-CMR with a supine ergometer overcomes this issue, but CMR scanning during exercise results in increased physical movement, respiratory artefacts and ECG gating artefacts, all of which increase with increasing workload [14]. Originally, Ex-CMR studies, using retrospective cardiac gating, performed imaging during exercise cessation and breath holding to overcome these issues [15], unfortunately both are non-physiological and reduce clinical utility. Progression to real-time imaging allowed free breathing during Ex-CMR [16]. The continued need for cardiac gating resulted in detrimental artefacts at maximal exercise and real time Ex-CMR studies assessing flow report the acquisition of a significant volume of flow data (< 25,000 images per patient), requiring the use of an online graphics processing unit reconstruction system and prolonged post processing/analysis time [17]. The development of un-gated real-time cine imaging solved the ECG gating issues, allowing biventricular volume assessment during maximal exercise [14]. Recently, combining this technique with un-gated flow acquisition resulted in the first study assessing bi-ventricular volumes and aortic and pulmonary flow during continuous exercise [18]. Unfortunately, the un-gated real-time technique requires specialist software (for post hoc cardiac and respiratory gating) and prolonged post processing and analysis time, thus decreasing clinical utility and widespread attainability. Compressed SENSE (C-SENSE) is a novel parallel imaging technique, robust to respiratory motion and allows fast image acquisition whilst maintaining high image quality [19]. To our knowledge [20], C-SENSE has not previously been utilised in Ex-CMR. The aims of this study are to demonstrate the feasibility of assessing biventricular volume and flow during continuous exercise using vendor provided C-SENSE sequences and commercially available standard analysis software.

## Materials and methods

### Design

Protocol development and feasibility testing was achieved by: (1) developing a free-breathing C-SENSE protocol and validating this against our institute’s standard clinical imaging sequences at rest; (2) determining the optimal acceleration of C-SENSE for PCMR sequences, for use in Ex-CMR, by assessing resting and exercise image quality and comparing the derived stroke volumes against standard clinical imaging sequences at rest; (3) utilising the validated C-SENSE protocol during continuous low and moderate exercise intensities to determine if the acquired biventricular volumes and flow have internal validity in terms of consistency of ventricular stroke volumes when derived separately from cavity volumes and great vessel flow measurements, and whether they are concordant with expected supine exercise physiology.

This study was approved by a local ethics committee in England (Yorkshire and the Humber—Leeds East 18/YH/0168). All participants provided written informed consent. All Ex-CMR studies were performed at the Leeds General Infirmary, UK.

### Study population

12 healthy volunteers (8 male, 4 female), aged 35 ± 9 years (mean ± standard deviation) (range 23–56 years) underwent CMR at rest and during continuous exercise using the Lode BV supine bicycle ergometer. Participants were of a healthy weight (BMI 23.9 ± 2.3) and of varying levels of physical fitness, performing regular exercise between 0.5 and 15 h a week (mean 5.0 ± 3.5 h). All healthy volunteers had no significant co-morbidities and no contraindications to exercise testing as per American heart association guidelines [21].

### Exercise protocol

**Fig. 1:**
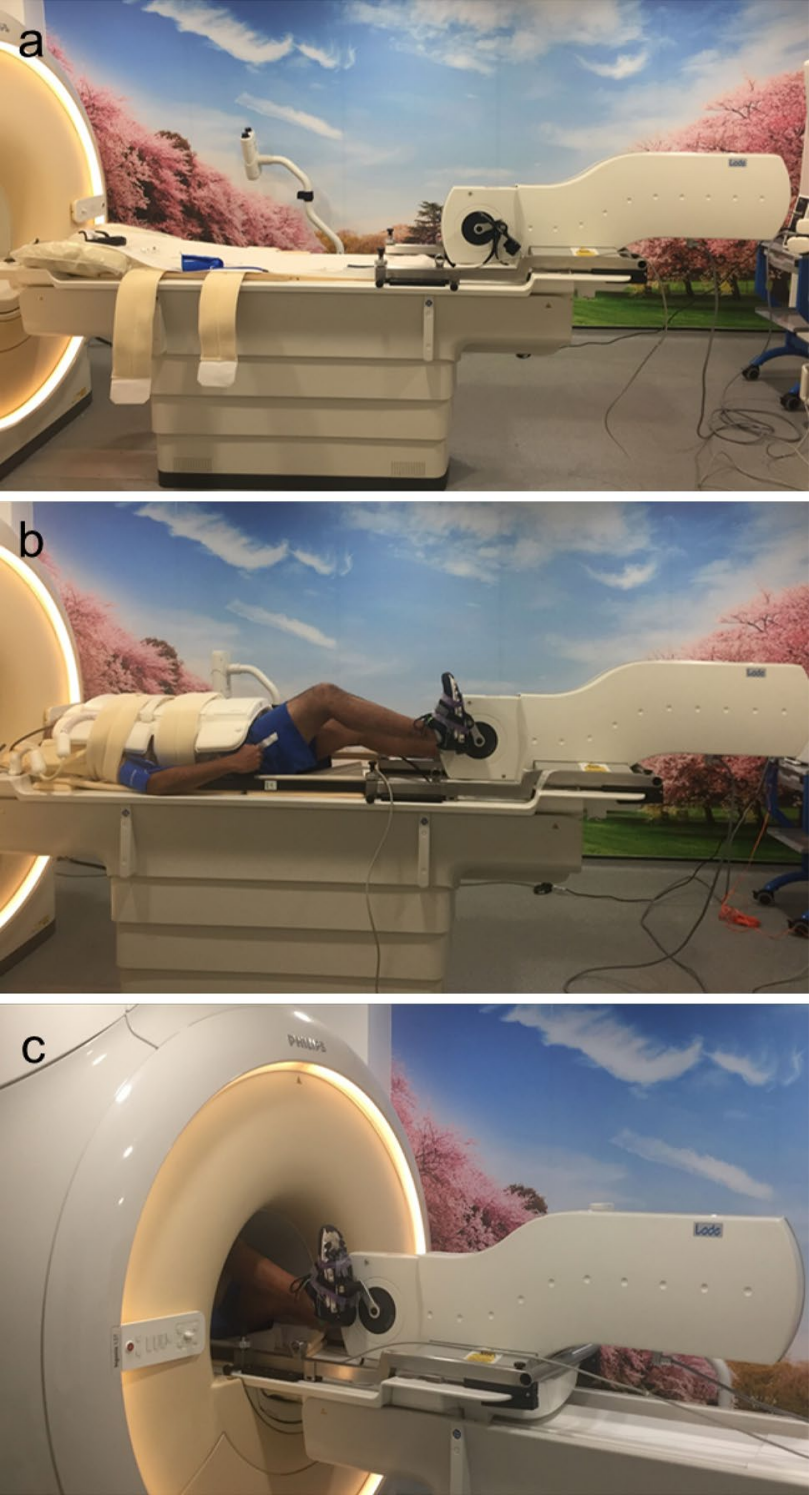
The Lode BV supine cycle ergometer before (**a**), during set up (**b**) and during use (**c**)

Participants performed supine cycle ergometer (Lode BV, Netherlands) (Fig. 1) exercise during CMR using heart rate reserve (HRR) and an age predictive maximal heart rate model [22], to prescribe individualised low (30–39% HRR) and moderate (40–59% HRR) exercise intensities. After completion of resting imaging, participants exercised with no resistance, 0 Watts (W), for 1 min at a cycling cadence of 60–70 rpm (with verbal feedback given to maintain this) then at an increase of 25 W every 2 min until ‘low intensity’ target heart rate (THR) was achieved; once THR was achieved smaller alterations in resistance wattage were made to maintain THR. HR was stabilised for 30 s prior to initiating imaging. After completion of imaging at low exercise intensity, resistance was increased by 25 W initially and every 2 min until the prescribed moderate intensity was reached and HR stabilised for 30 s prior to initiating imaging. Exercise performed was continuous and all exercise imaging acquired during free-breathing, with the use of straps around the patient and receiver coil to reduce exercise motion artefact (Fig. 1). Participants perceived rate of exertion were assessed on the Borg scale after exercise cessation, to ensure correlation with prescribed intensity [23].

### CMR imaging

CMR imaging was performed on a dedicated cardiovascular 1.5T MR system (Philips Ingenia system, Best, Netherlands). Initial survey and cine imaging was performed including: vertical long axis, horizontal long axis, left ventricular outflow tract (LVOT) and right ventricular outflow tract (RVOT) views. At rest, our institute’s standard clinical protocol to assess biventricular volumes, aortic and pulmonary flow was performed to validate the novel C-SENSE protocol against. The C-SENSE protocol was used at rest and during continuous exercise to low and moderate intensities.

### Standard clinical protocol

Biventricular function was assessed using a breath-hold multi-phase, multi-slice short axis cine imaging stack (10 mm, no gap, 30 phases, SENSE 2). Great vessel flow was assessed from aortic and pulmonary through-plane phase contrast velocity mapping acquired during breath-hold (SENSE 2) and a separate free-breathing acquisition (no parallel imaging) to ensure a comprehensive comparison with the novel C-SENSE protocol.

### C-SENSE protocol

The evaluation protocol involved biventricular function assessment by free-breathing, respiratory navigated, continuous cine imaging in short axis geometry (10 mm, no gap) accelerated by a C-SENSE factor of 3 (CS3). Great vessel flow was assessed by aortic and pulmonary through-plane phase-contrast imaging, with two separate free-breathing acquisitions using C-SENSE 3 (CS3) and C-SENSE 6 (CS6) acceleration. CS3 and CS6 flow acquisitions were acquired to investigate if a higher acceleration would result in better image quality as a faster acquisition may be less prone to respiratory artefact. Additional CMR imaging parameters are described below.

During exercise, the above evaluation C-SENSE protocol was used with the addition of free-breathing LVOT/RVOT cine imaging being performed to assess for movement during exercise and re-plan the phase contrast imaging geometry if required.

### CMR imaging parameters

All image acquisitions, including cine imaging and PCMR imaging, were retrospectively cardiac gated. The clinical short axis cine imaging parameters were as follows: typical FOV 360 × 300 mm, TR 3.1msec, TE 1.56msec, flip angle 60°, SENSE factor 2, multishot TFE factor 12, TFE acquisition duration 37.4 ms, phase percentage 67%, slice thickness 10 mm, 0 mm gap, 30 phases, in-plane spatial resolution acquired at 1.88 × 1.88 mm and reconstructed to 1.25 × 125 mm, matrix 192 × 158, planned acquisition involved 7 × 8-s breath-holds. The C-SENSE short axis cine imaging parameters were as follows: typical FOV 300 × 300 mm, TR 2.4 msec, TE 1.21 msec, flip angle 60°, temporal resolution 32msec. C-SENSE factor 3, multishot TFE factor 13, TFE acquisition duration 31.5 ms, phase percentage 67%, slice thickness 10 mm, 0 mm gap, in-plane spatial resolution acquired at 2.5 × 2.5 mm and reconstructed to 1.34 × 1.34 mm, matrix 120 × 120, planned acquisition time 39 s. Respiratory navigation was used with the respiratory echo-based navigator positioned on the right hemi-diaphragm using a 5 mm acceptance window with continuous gating level drift.

**Fig. 2:**
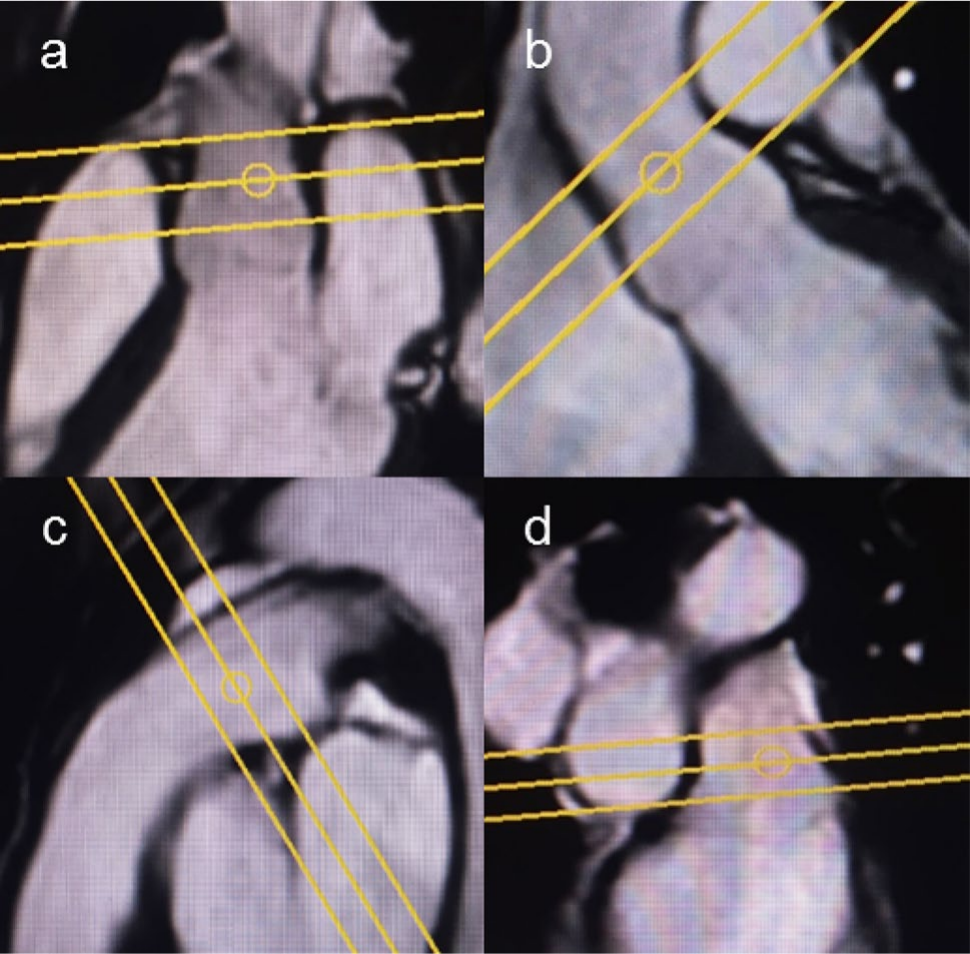
Example of planning of aortic (**a**, **b**) and pulmonary (**c**, **d**) PCMR-stack. Aortic flow stack geometry, 8 mm slices with -3 mm slice gap. Pulmonary PCMR-stack geometry, 8 mm slices with -5 mm slice gap. **a** planning of aortic PCMR-stack in LVOT1 geometry. **b** planning of aortic PCMR-stack in LVOT2 geometry. **c** planning of pulmonary PCMR-stack in RVOT1 geometry. **d** planning of pulmonary PCMR-stack in RVOT2 geometry

Through-plane velocity encoded (VENC) PCMR was acquired at the aortic sino-tubular junction for aortic PCMR and in the main pulmonary artery (MPA) 1 cm superior to the valve for pulmonary PCMR. Resting VENC was set to 150 cm/s and increased to 250 cm/s during exercise; the VENC was increased further if aliasing occurred. To accommodate for potential through-plane motion during exercise, the CS3 and CS6 PCMR sequences were performed using a novel ‘PCMR-imaging stack’ acquiring 3 × 8 mm overlapping PC-slices orthogonal to vessel flow (Fig. 2). Aortic PCMR sequences used a − 3 mm gap (thus the centre of the slices are spaced 5 mm apart) and the pulmonary flows had − 5 mm gap (thus the centre of the slices are spaced 3 mm apart).The increased overlap of the pulmonary PCMR sequences was to accommodate for the short length of the main pulmonary artery prior to bifurcation, which has led to difficulty performing pulmonary PCMR in previous Ex-CMR studies [24].

The clinical breath held (SENSE 2) and free-breathing CS3 and CS6 gradient echo PCMR sequences shared the following imaging parameters: typical FOV 350 × 320 mm, TR 4.9msec, TE 2.9 msec, flip angle 15°, number of signal averages 1, turbo field echo (TFE) factor 4, slice thickness 8 mm, 30 phases, phase percentage 67%, acquired in-plane spatial resolution 2.5 × 2.5 mm reconstructed to 1.22 × 1.22 mm, matrix 140 × 128, Cartesian sampling, planned acquisition time (per slice) of 13, 9 and 5 s for SENSE-2, CS3 and CS6 PCMR sequences respectively. The imaging parameters of the free-breathing standard clinical sequence (with no parallel imaging) were as follows: typical FOV 400 × 280 mm, TR 17 msec, TE 2.4 msec, flip angle 40°, number of signal averages 1, slice thickness 6 mm, 40 phases, in-plane spatial resolution 1.56 × 2.23 mm, matrix 256 × 126, Cartesian sampling, typical acquisition duration: 101 s.

### CMR analysis

Images were analysed using commercially available software (cvi42, Circle Cardiovascular Imaging, Calgary, AB, Canada). LV and RV endocardial contours were manually traced with the papillary muscles and trabeculations considered part of the ventricular blood pool and volumes calculated by summation of disks. Aortic and pulmonary flows were assessed by manually contouring the vessel in every phase. The CS3/CS6 PCMR-stack was assessed for the slice closest resembling the resting standard clinical acquisition to ensure all PCMR images had flow assessed at the same anatomical level. Image quality assessment was performed on all assessed PCMR images independently by two assessors (TC & NJ), whom were blinded to each-others results. Images were graded on the following scale: 3- excellent, 2- good, 1- adequate & 0- non diagnostic; the mean image quality scores from both assessors are presented.

### Statistical analysis

Data were analysed using SPSS version 26 (IBM Corp.) and Microsoft Excel 2010. All continuous data were assessed for normality using Shapiro–Wilk test. Resting biventricular parameters comparing the breath-held standard clinical with CS3 respiratory navigated SA acquisitions were assessed by Pearsons correlation and the bias and limits of agreement by Bland–Altman plots. PCMR image quality scores were assessed by Wilcoxon signed ranks test and the stroke volume comparisons assessed by repeated measures ANOVA with Bonferroni post-test analysis. Repeated measures ANOVA with Bonferroni post-test analysis was used to compare cardiac volumetric and flow data between rest and different stages of exercise. Intra-observer analysis was performed by TC and inter-observer analysis by NJ; reproducibility was assessed by the Coefficient of Variation (CV) test, the standard deviation of differences between observations divided by the mean and by intra-class correlation (ICC) with a two way random model for absolute agreement. p < 0.05 was considered statistically significant. Intra and inter-observer analysis was performed in a blinded method.

## Results

13 healthy volunteers completed the study protocol, 1 volunteer was excluded due to ECG gating issues at moderate exercise intensity, leaving 12 healthy volunteers for analysis (8 male, age 35 ± 9 years, BMI 23.9 ± 2.3 kg/m^2^).

### Validation of free-breathing C-SENSE protocol at rest

**Fig. 3:**
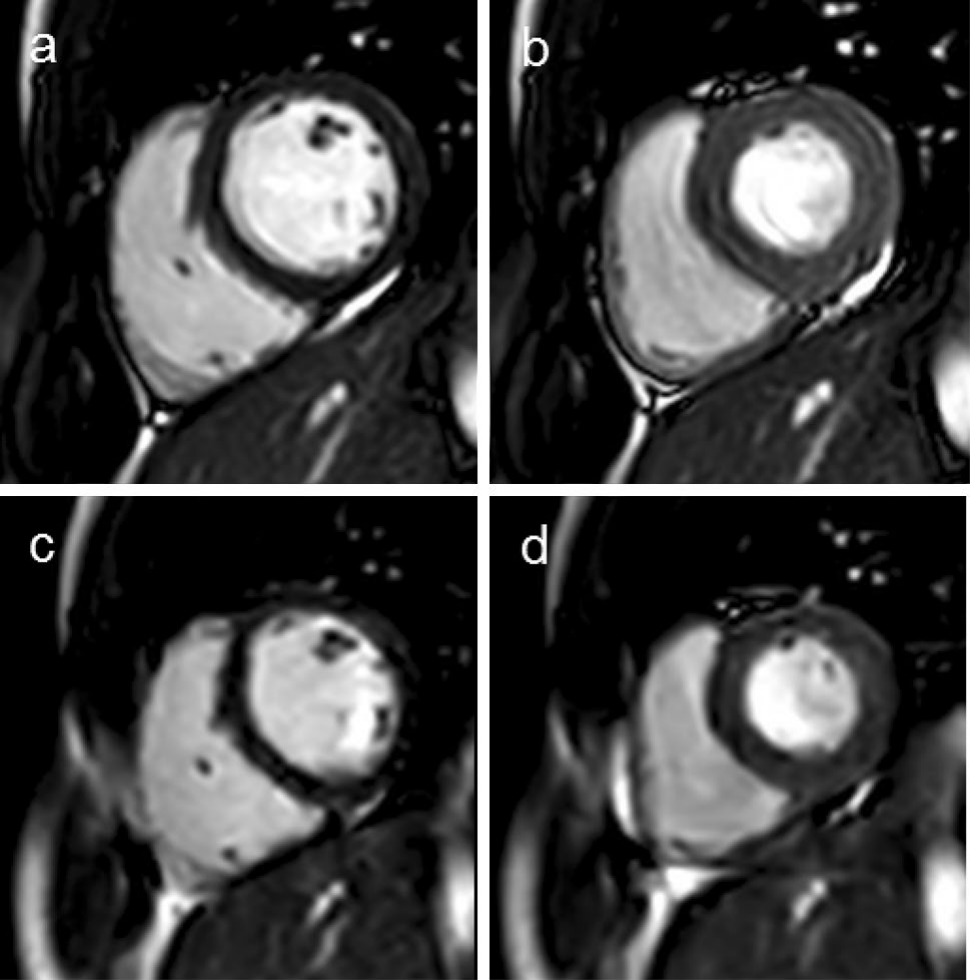
Image quality comparison of resting short axis images acquired by clinical breath held SENSE 2 sequences at end-diastole (**a**) and end-systole (**b**) and Compressed SENSE 3 respiratory navigated sequences at and end-diastole (**c**) and end-systole (**d**)

At rest, there were no significant differences between the biventricular volumes assessed by the standard clinical or novel CS3 short axis sequences, with all parameters demonstrating minimal bias and very strong correlation (r > 0.93, p ≤ 0.01) (Table 1). Figure 3 demonstrates the typical image quality comparison between the resting breath-hold standard clinical and free-breathing CS3 short axis sequences. Mean resting aortic and pulmonary stroke volumes acquired from all 4 PCMR sequences were comparable, with CS3 and CS6 free-breathing flow showing minimal bias with both breath-hold and free-breathing standard clinical flow sequences (Table 2). However, CS6 aortic flow measurements were more prone to underestimate aortic flow, with a bias of − 2.15 ml/m^2^/cardiac cycle against the breath-hold clinical standard in comparison to a minimal bias of − 0.12 ml/m^2^/cardiac cycle using a CS3 flow sequence. Additionally, pulmonary stroke volumes from CS6 sequences only demonstrated moderate correlation with clinical free-breathing sequences (r = 0.655).

**Table 1:** Validation of compressed SENSE 3 free-breathing sequences at rest vs breath-held clinical standard

Measurement	Image sequence		Bland altman				Correlation coefficient	
	Clinical	CS3	RC	Upper	Lower	Bias	r	p-value
LVEDV (ml)	165 ± 39	164 ± 39	7.05	6.34	− 7.76	− 0.71	0.996	< 0.01
LVEDVi (ml/m^2^)	88.8 ± 16	88.5 ± 16	3.69	3.33	− 4.05	− 0.36	0.994	< 0.01
LVESV (ml)	73 ± 23	71 ± 23	10.38	9.46	− 11.29	− 0.92	0.976	< 0.01
LVESVi (ml/m^2^)	38.9 ± 10	38.4 ± 11	5.42	4.95	− 5.88	− 0.46	0.971	< 0.01
LVSV (ml)	92 ± 19	93 ± 19	6.38	6.58	− 6.18	0.2	0.986	< 0.01
LVSVi (ml/m^2^)	50 ± 7	50 ± 7	3.35	3.46	− 3.24	0.11	0.974	< 0.01
LVEF (%)	57 ± 6	57 ± 6	4.74	5.2	− 4.28	0.46	0.932	< 0.01
RVEDV (ml)	166 ± 36	166 ± 34	8.59	9.21	− 7.96	0.62	0.995	< 0.01
RVEDVi (ml/m^2^)	89.4 ± 16	89.8 ± 15	4.74	5.15	− 4.34	0.41	0.991	< 0.01
RVESV (ml)	75 ± 24	75 ± 21	7.27	6.78	− 7.76	− 0.49	0.992	< 0.01
RVESVi (ml/m^2^)	40.6 ± 11	40.4 ± 10	3.84	3.6	− 4.07	− 0.23	0.99	< 0.01
RVSV (ml)	90 ± 18	91 ± 17	6.46	7.56	− 5.35	1.1	0.985	< 0.01
RVSVi (ml/m^2^)	48.8 ± 8	49.4 ± 7	3.51	4.15	− 2.88	0.63	0.977	< 0.01
RVEF (%)	55 ± 7	56 ± 6	2.82	3.23	− 2.42	0.4	0.985	< 0.01
Aortic SV (ml)	89 ± 18	89 ± 17	7.75	7.44	− 8.05	− 0.31	0.978	< 0.01
Pulmonary SV (ml)	90 ± 15	89 ± 18	12.42	11.21	− 13.63	-1.21	0.944	< 0.01

*CS3* Compressed SENSE 3, *EDV* end-diastolic volume, *EF* ejection fraction, *ESV* end-systolic volume, *HR* heart rate, *i* indexed to body surface area, *LV* left ventricle, *RC* repeatability coefficient, *RV* right ventricle, *SV* stroke volume

**Table 2:** Comparisons of resting stroke volumes indexed to BSA between clinical and C-SENSE accelerated aortic and pulmonary flow sequences

	SV (ml/m^2^)	Flow comparison			
		Vs Clinical BH		Vs Clinical FB	
		Bias (ml/m^2^)	CC (r)	Bias (ml/m^2^)	CC (r)
Aortic flow					
BH	48.3 ± 7.1	–	–	0.11	0.762
FB	48.4 ± 5.7	− 0.11	0.762	–	–
CS3	48.2 ± 6.7	− 0.12	0.96	− 0.23	0.865
CS6	46.2 ± 6.4	− 2.15	0.849	− 2.26	0.873
Pulmonary flow					
BH	48.9 ± 5.9	–	–	0.53	0.8
FB	48.3 ± 6.4	− 0.53	0.8	–	–
CS3	48.2 ± 7.6	− 0.69	0.915	− 0.16	0.909
CS6	48.1 ± 6.6	− 0.73	0.85	− 0.20	0.655

*BH* breath hold, *CC* correlation coefficient (Pearsons), *CS3/CS6* compressed SENSE 3/6, *FB* free-breathing, *SV* stroke volume

### Image quality scoring

**Fig. 4:**
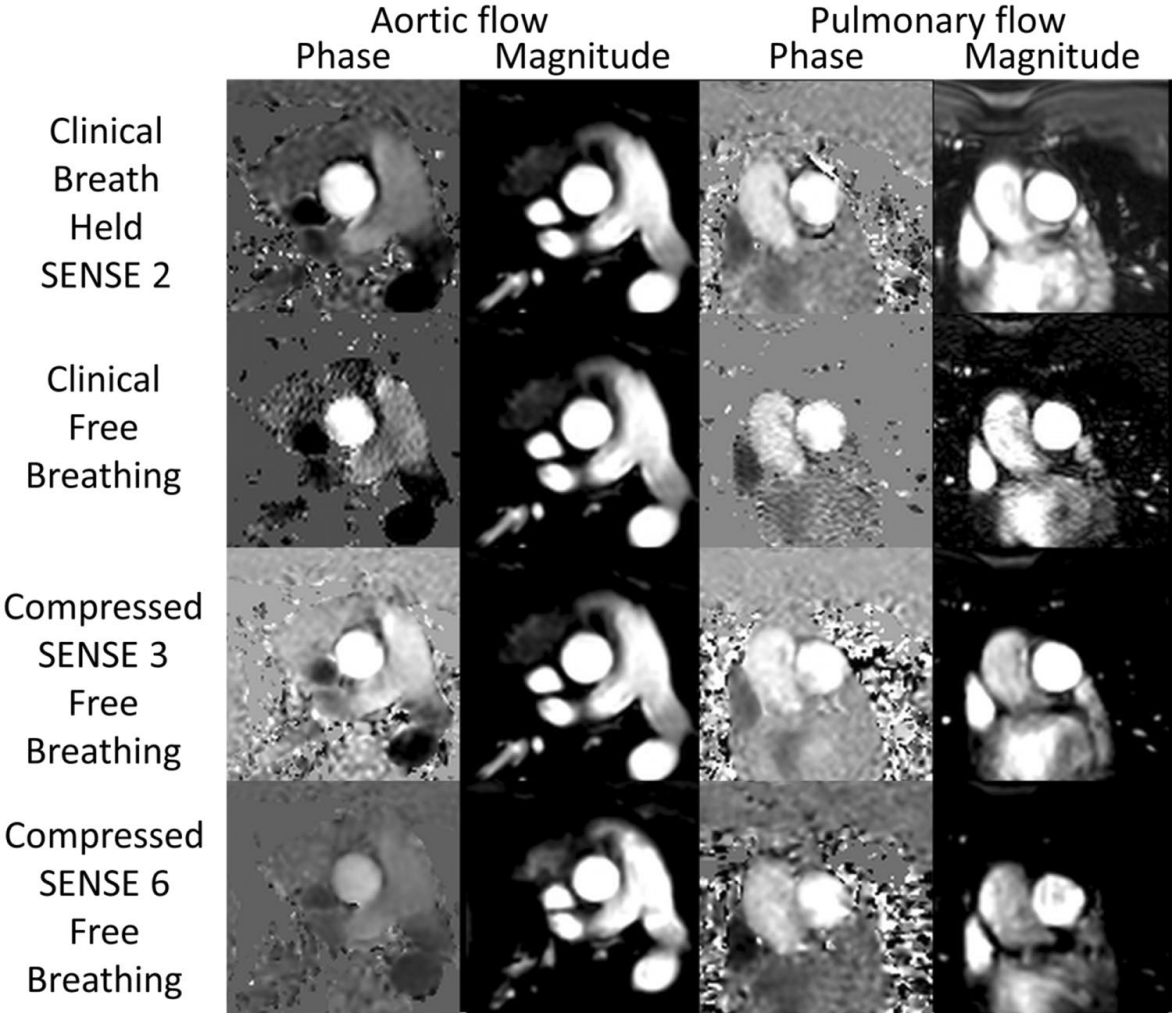
Image quality of resting phase contrast image sequences, comparing both clinical standard sequences (Clinical breath held SENSE 2 & clinical free-breathing no parallel imaging) with Compressed SENSE 3 and Compressed SENSE 6 sequences

As expected, resting clinical breath-hold image quality scores for aortic and pulmonary flows were significantly higher compared to free-breathing sequences (p < 0.01), except when compared with CS3 pulmonary flow (p = 0.06) (Table 3). At rest, CS3 flow sequences had the highest image quality scores of all free-breathing sequences, including the free-breathing clinical sequence, and were significantly greater than CS6 sequences for aortic (p = 0.02) and pulmonary (p < 0.01) flow. Figure 4 demonstrates the image quality of the different resting flow images acquired in the same patient. During exercise the image quality scores of CS3 aortic and pulmonary flow sequences were consistently higher than CS6 flow sequences. Indeed at moderate exercise intensity, five aortic and two pulmonary flow CS6 sequences were considered non-diagnostic, whereas all CS3 flow sequences were of adequate diagnostic quality. Due to the non-diagnostic image quality described in numerous CS6 flow acquisitions at moderate exercise intensity, the CS6 flow sequences were deemed unsuitable for Ex-CMR flow assessment and future studies.

**Table 3:** Image quality score comparison between flow sequences at rest and exercise

Flow sequence	Resting		Low exercise		Moderate exercise	
	Aortic	Pulmonary	Aortic	Pulmonary	Aortic	Pulmonary
Clinical BH	2.83 ± 0.24*^+#^	2.88 ± 0.30*^#^	–	–	–	–
Clinical FB	2.21 ± 0.38	2.08 ± 0.45^#^	–	–	–	–
CS3 free-breathing	2.33 ± 0.3^#^	2.38 ± 0.58^#^	1.5 ± 0.41^#^	1.46 ± 0.62^#^	1.21 ± 0.25^#^	1.08 ± 0.19
CS6 free-breathing	1.75 ± 0.32	1.63 ± 0.30	1.33 ± 0.37	1.13 ± 0.22	0.88 ± 0.46	0.88 ± 0.41

Image quality score: 3- excellent, 2- good, 1- adequate & 0- non diagnostic

*p ≤ 0.05 superior to clinical free-breathing sequence at same exercise stage, ^+^p ≤ 0.05 superior to CS3 sequence at same exercise stage, ^#^p ≤ 0.05 superior to CS6 sequence at same exercise stage. *BH* breath held, *CS* compressed SENSE, *FB* free-breathing

### Supine bicycle exercise

The participants’ haemodynamic responses to supine bicycle exercise are displayed in Table 4. Participants’ maintained within the target HR during each exercise stage increasing from 58 ± 6 bpm at rest, to 102 ± 5 bpm and 119 ± 5 bpm at low and moderate exercise respectively. Systolic BP rose with increasing exercise intensity (119 ± 10 mmHg at rest to 143 ± 15 mmHg at low and 160 ± 24 mmHg at moderate exercise), whilst diastolic BP remained unchanged (71 ± 8 mmHg at rest to 76 ± 13 mmHg at low and 75 ± 13 mmHg at moderate exercise). BP was un-recordable at moderate exercise intensity in two subjects. Participants subjective rating of perceived exertion (RPE) on the Borg scale [23] were 9.6 ± 1.8 for low and 13.7 ± 2.4 for moderate exercise intensities, falling into the target ranges, as per ACSM guidelines [25], for the prescribed exercise intensities. Therefore, both the objective haemodynamic and the subjective Borg RPE scores were within the advised ranges for the prescribed exercise intensities.

**Table 4:** Haemodynamic response to supine bicycle exercise using the C-SENSE 3 protocol

	Rest	Low	Moderate	ANOVA P-value	Rest vs Low	Low vs Mod	Rest vs Mod
HRR % Of HR_max_	N/A	30–39%	40–59%	–	–	–	–
HR achieved	58 ± 6	102 ± 5	119 ± 5	< 0.001	< 0.001	< 0.001	< 0.001
Systolic BP*	119 ± 10	143 ± 15	160 ± 24	< 0.001	0.001	0.038	< 0.001
Diastolic BP*	71 ± 8	76 ± 13	75 ± 13	0.605	1	1	1
Borg RPE	6 ± 0	9.6 ± 1.8	13.7 ± 2.4	< 0.001	< 0.001	< 0.001	< 0.001
Cycle resistance (W)	0	52 ± 26	84 ± 24	< 0.001	< 0.001	< 0.001	< 0.001
LVEDV (ml)	164 ± 39	163 ± 36	159 ± 34	0.052	1	0.187	0.192
LVEDVi (ml)	88.5 ± 16	88.2 ± 15	85.9 ± 14	0.066	1	0.173	0.256
LVESV (ml)	71 ± 23	58 ± 21	49 ± 20	< 0.001	0.001	0.001	< 0.001
LVESVi (ml)	38.4 ± 11	31 ± 10	26.4 ± 10	< 0.001	0.001	0.001	< 0.001
LVSV (ml)	93 ± 19	106 ± 19	110 ± 19	< 0.001	0.002	0.193	< 0.001
LVSVi (ml)	50 ± 7	57.2 ± 8	59.5 ± 7	< 0.001	0.002	0.177	< 0.001
LVEF (%)	57 ± 6	66 ± 7	70 ± 8	< 0.001	< 0.001	0.002	< 0.001
Aortic SV (ml)	89 ± 17	102 ± 18	105 ± 18	< 0.001	0.001	0.708	< 0.001
Aortic SVi (ml)	48.2 ± 7	55.1 ± 8	56.6 ± 8	< 0.002	0.001	0.682	< 0.001
RVEDV (ml)	166 ± 34	161 ± 33	158 ± 31	0.003	0.104	0.18	0.025
RVEDVi (ml)	89.8 ± 15	87.2 ± 15	85.2 ± 14	0.002	0.096	0.16	0.023
RVESV (ml)	75 ± 21	58 ± 20	48 ± 17	< 0.001	< 0.001	0.001	< 0.001
RVESVi (ml)	40.4 ± 10	31.1 ± 10	25.8 ± 8	< 0.001	< 0.001	0.001	< 0.001
RVSV (ml)	91 ± 17	104 ± 18	110 ± 17	< 0.001	< 0.001	0.008	< 0.001
RVSVi (ml)	49.4 ± 7	56.1 ± 7	59.4 ± 7	< 0.001	< 0.001	0.008	< 0.001
RVEF (%)	56 ± 6	65 ± 7	70 ± 6	< 0.001	< 0.001	< 0.001	< 0.001
Pulmonary SV (ml)	89 ± 18	100 ± 17	102 ± 16	< 0.001	0.007	1	0.012
Pulmonary SVi (ml)	48.2 ± 8	54.3 ± 7	55.2 ± 7	< 0.001	0.005	1	0.009

*BP* blood pressure, *EDV* end-diastolic volume, *EF* ejection fraction, *ESV* end-systolic volume, *HR* heart rate, *i* indexed to body surface area, *LV* left ventricle, *RPE* rate of perceived exertion, *RV* right ventricle, *SV* stroke volume

*Blood pressure was unrecordable in 2 patients at moderate exercise intensity

### Cardiac indices response to exercise

#### Volumes

**Fig. 5:**
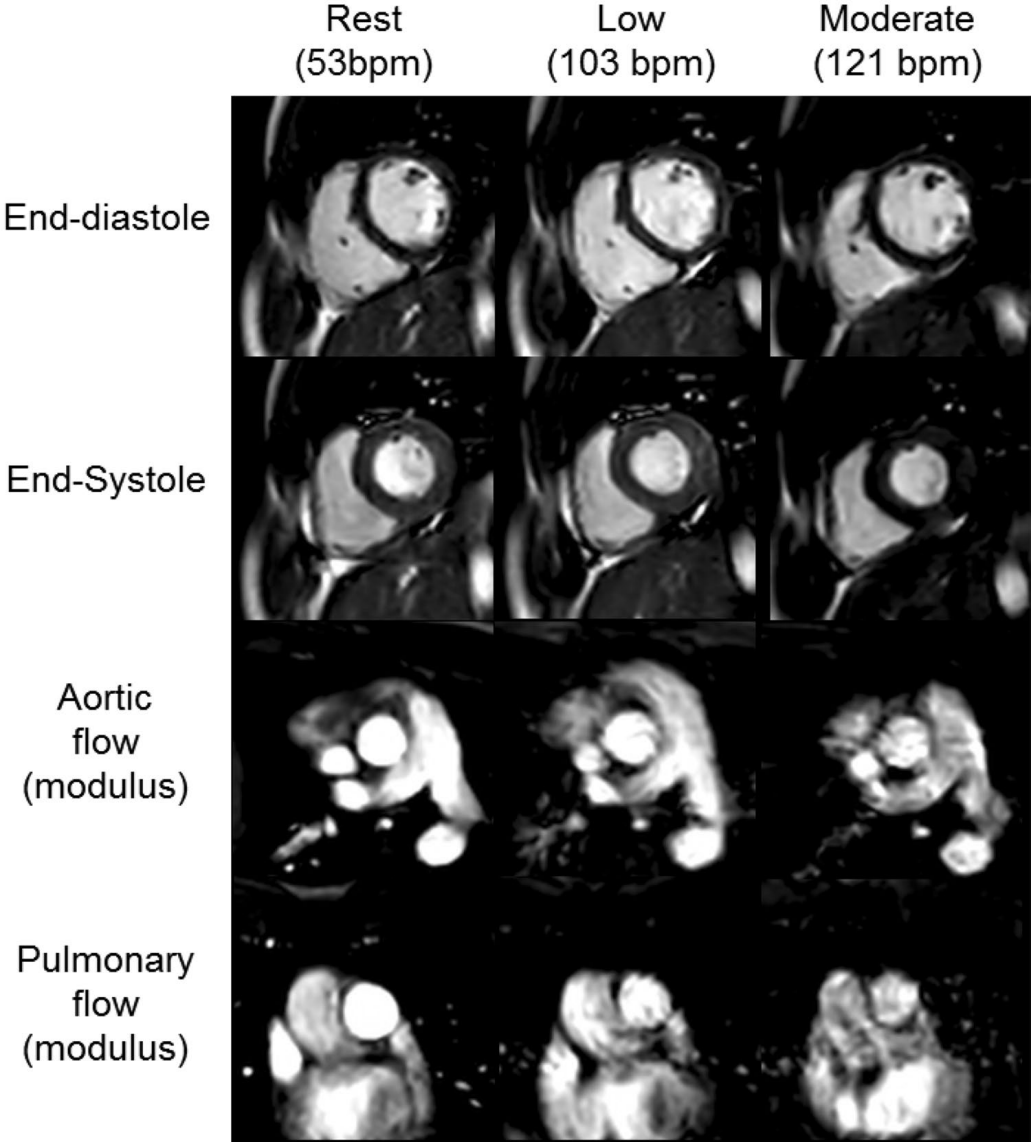
Typical image quality of cine and phase contrast imaging at rest and during Ex-CMR to low and moderate exercise

Table 4 demonstrates the cardiac volumetric and flow changes during exercise and Fig. 5 shows the typical image quality obtained during exercise for both cine and aortic and pulmonary PCMR images. During Ex-CMR, indexed left ventricular end-diastolic volume (LVEDVi) did not significantly alter (88.5 ± 16 ml/m^2^ at rest, 88.2 ± 15 ml/m^2^ at low an 85.9 ± 14 ml/m^2^ at moderate, p = 0.256 for rest to moderate exercise), indexed LV stroke volume (LVSVi) increased significantly (50 ± 7 ml/m^2^ at rest, 57.2 ± 8 ml/m^2^ at low and 59.5 ± 7 ml/m^2^ at moderate exercise; p ≤ 0.001 for rest to moderate exercise) driven by a significant fall in indexed LV end-systolic volume (LVESVi) (38.4 ± 11 ml/m^2^ at rest vs 31 ± 10 ml/m^2^ at low and 26.4 ± 10 ml/m^2^ at moderate; p ≤ 0.001 for rest to moderate exercise) thus causing a rise in LV ejection fraction (LVEF) with exercise (57 ± 6% at rest, 66 ± 7% at low and 70 ± 8% at moderate exercise; p ≤ 0.001 for rest to moderate exercise). During Ex-CMR, right ventricular end-diastolic volume (RVEDVi) decreased significantly (89.8 ± 15 ml/m^2^ at rest, 87.2 ± 15 ml/m^2^ at low and 85.2 ± 14 ml/m^2^ at moderate exercise, p = 0.023 rest to moderate exercise), indexed right ventricular end-systolic volume (RVESVi) decreased (40.4 ± 10 ml/m^2^ at rest vs 31.1 ± 10 ml/m^2^ at low and 25.8 ± 8 ml/m^2^ at moderate exercise; p ≤ 0.001 for rest to moderate exercise) driving a rise in indexed right ventricular stroke volume (RVSVi) (49.4 ± 7 ml/m^2^ at rest, 56.1 ± 7 ml/m^2^ at low and 59.4 ± 7 ml/m^2^ at moderate; p ≤ 0.001 for rest to moderate exercise) and right ventricular ejection fraction (RVEF) (56 ± 6% vs 65 ± 7% at low and 70 ± 6% at moderate exercise; p =  < 0.001 for rest to moderate exercise) with increasing exercise.

#### Flow

Aortic stroke volumes increased significantly during Ex-CMR from 48.2 ± 7 ml/m^2^/cardiac cycle at rest to 55.1 ± 8 ml/m^2^/cardiac cycle at low and 56.6 ± 8 ml/m^2^/cardiac cycle at moderate exercise intensities (p ≤ 0.001, rest to moderate exercise). Aortic stroke volumes showed very strong correlation with LVSVi at rest (r = 0.93), low (r = 0.97) and moderate exercise (r = 0.98). During Ex-CMR pulmonary stroke volumes increased significantly from 48.2 ± 8 ml/m^2^/cardiac cycle at rest to 54.3 ± 7 ml/m^2^/cardiac cycle at low and 55.2 ± 7 ml/m^2^/cardiac cycle at moderate exercise intensities (p = 0.009, rest to moderate exercise) and correlated strongly with RVSVi at rest (r = 0.88) and very strongly during low (r = 0.90) and moderate exercise (r = 0.97).

### Intra/inter-observer reproducibility

Intra- and inter-observer reproducibility is shown in Table 5. Intra-observer reproducibility of all cardiac sequences assessed at rest and during exercise by CV were excellent (CV < 10%) and all sequences assessed by ICC were excellent (ICC > 0.9) with exception of pulmonary flow at low (ICC = 0.892) and moderate exercise (ICC = 0.847) and LVSV at moderate exercise (ICC = 0.897).

**Table 5:** Coefficient of Variation and intra-class correlation coefficient for the reproducibility of biventricular volumetric and flow indices

Exercise level and sequence	Cardiac parameter	Intra-observer		Inter-observer	
		COV	ICC	COV	ICC
Resting clinical	LVEDV	1.32	0.996	2.12	0.988
	LVESV	2.69	0.989	6.58	0.968
	LVSV	2.47	0.967	4.24	0.920
	LVEF	2.06	0.987	3.75	0.931
	RVEDV	2.29	0.985	2.65	0.979
	RVESV	5.34	0.968	8.60	0.918
	RVSV	3.94	0.953	6.89	0.808
	RVEF	3.55	0.957	6.35	0.877
	Aortic flow FB	1.14	0.990	3.07	0.930
	Aortic flow BH	0.83	0.997	2.05	0.980
	Pulmonary flow FB	1.18	0.993	2.15	0.973
	Pulmonary flow BH	1.40	0.988	1.78	0.981
Resting compressed SENSE 3 free-breathing	LVEDV	1.29	0.995	2.50	0.985
	LVESV	3.89	0.976	6.66	0.965
	LVSV	2.89	0.958	3.44	0.942
	LVEF	2.98	0.974	3.41	0.953
	RVEDV	1.92	0.986	4.15	0.937
	RVESV	5.40	0.957	12.96	0.814
	RVSV	3.67	0.945	6.24	0.824
	RVEF	3.23	0.968	7.26	0.817
	Aortic flow	0.83	1.000	1.19	0.993
	Pulmonary flow	2.19	0.986	3.55	0.950
Low intensity exercise (CS3 free-breathing)	LVEDV	0.76	0.998	3.97	0.953
	LVESV	8.77	0.915	11.38	0.911
	LVSV	4.44	0.907	3.46	0.952
	LVEF	4.72	0.923	4.08	0.916
	RVEDV	1.95	0.984	3.72	0.955
	RVESV	8.78	0.907	11.39	0.909
	RVSV	2.97	0.947	3.07	0.940
	RVEF	4.37	0.934	4.09	0.908
	Aortic flow	1.99	0.986	5.88	0.917
	Pulmonary flow	3.13	0.892	3.84	0.927
Moderate intensity exercise (CS3 free-breathing)	LVEDV	2.09	0.986	4.27	0.940
	LVESV	9.50	0.952	16.61	0.883
	LVSV	4.17	0.897	4.37	0.849
	LVEF	3.54	0.956	4.96	0.891
	RVEDV	3.48	0.964	5.77	0.878
	RVESV	9.23	0.926	17.93	0.754
	RVSV	3.77	0.923	4.99	0.830
	RVEF	2.95	0.955	5.12	0.837
	Aortic flow	2.22	0.975	4.01	0.918
	Pulmonary flow	6.22	0.847	6.11	0.879

*COV* co-efficient of variance, *EDV* end-diastolic volume, *EF* ejection fraction, *ESV* end-systolic volume, *HR* heart rate, *i* indexed to body surface area, *ICC* intra-class correlation, *LV* left ventricle, *RV* right ventricle

Inter-observer reproducibility assessed by CV of cardiac parameters were similarly excellent (CV < 10%), with the exception of RVESV by CS3 cine imaging at rest (CV 12.96%) and LVESV and RVESV during exercise, with a CV of 11.38% and 11.39% at low and 16.61% and 17.93% at moderate exercise intensities respectively. Cardiac parameters demonstrated excellent ICC (> 0.9) at rest with the exception of RVSV & RVEF on clinical sequences and RVESV, RVSV & RVEF on CS3 sequences demonstrating good ICC (> 0.8). During low intensity exercise all cardiac parameters demonstrated excellent ICC (> 0.9), which decreased to good ICC at moderate exercise (ICC > 0.75) with the exception of LVEDV and aortic flow which maintained excellent ICC (> 0.9). The increase in variability of end-systolic volumes with increased exercise intensity is not unsurprising given the significant fall in ESV with exercise which allows for a smaller margin of error.

## Discussion

This study has shown that (1) it is feasible to assess biventricular volumes and flow by CMR during continuous in-scanner supine bicycle exercise using free-breathing C-SENSE, (2) Using CS3 compared to standard clinical imaging, image quality and reproducibility were good, but this was not the case with higher acceleration factors (CS6) and (3) Using CS3, we have shown superior reproducibility in comparison to the only previous study to perform biventricular volume and flow assessment during continuous Ex-CMR (which used un-gated real-time sequences) [18].

To our knowledge [20], only one prior study, by Jaijee et al., has assessed biventricular volume and flow assessment with free-breathing during continuous exercise, and did so by utilising an un-gated real-time technique [18]. The study was insightful, investigating right ventricular dysfunction in acute hypoxia and chronic pulmonary arterial hypertension. However the authors didn’t perform image quality assessment and demonstrated suboptimal reproducibility, on the basis of ICC for intra- and inter-observer variability for RVEF. Our RVEF ICC for intra- and inter-observer analysis respectively was 0.968 and 0.817 at rest, and 0.955 and 0.837 at moderate exercise (vs 0.71 and 0.85 at rest and 0.625 and 0.744 at moderate exercise in the un-gated real-time study). One caveat with this direct comparison is we only studied healthy volunteers, whereas Jaijee et al. studied healthy volunteers and patients with pulmonary hypertension [18]; patients may demonstrate increased respiratory motion, worse image quality and so a resultant decrease in reproducibility. Therefore our technique needs testing in patients with cardiac disease before direct comparisons can be confidently made. Both studies represent a significant progression in the potential clinical utility of Ex-CMR, however our study is the first study to demonstrate such feasibility using vendor provided sequences with analysis performed on standard commercially available software.

Comparatively lower heart rates are observed during supine exercise compared with upright exercise at the same intensity. Exercise in the supine position results in higher blood pressure than upright exercise [26], therefore a similar double product (systolic blood pressure x heart rate), which is an index of myocardial oxygen consumption [27], is achieved at lower heart rates than upright exercise [28–30]. Therefore, we used heart rate reserve (HRR) to determine subject specific target heart rates, with the resting heart rate assessed when supine. Importantly, our study aimed only to assess subjects to moderate exercise intensity, and not to submaximal or maximal intensity. Maximal in-scanner continuous exercise can create significant motion artefacts, rendering images non-diagnostic, but more importantly may be unsafe in a patient population, given the inability to accurately assess ST segment changes which could prompt test termination. However, even at moderate intensity exercise, an Ex-CMR protocol assessing biventricular function and flow, may theoretically provide additional diagnostic and prognostic information in valvular and congenital heart disease, especially for valvular regurgitation assessment.

The haemodynamic response to exercise demonstrated a minimal change in LVEDV and a rise in LVSV driven by a fall in LVESV during exercise, which is in keeping with a recent Ex-CMR meta-analysis of 16 Ex-CMR studies [31]. Indeed, our study demonstrated a non-significant decrease in LVEDV with exercise as was demonstrated by the majority of Ex-CMR studies in the Ex-CMR meta-analysis. These findings replicate the theory that being truly supine (rather than recumbent in stress echocardiography) results in near maximal LVEDV at rest and thus no significant increase is seen with exercise.

### Clinical implications

The clinical utility of Ex-CMR requires rapid image acquisition using accessible free-breathing sequences and analysis software. We demonstrated this is feasible using C-SENSE. C-SENSE is vendor provided, boasting faster image acquisition [32, 33] and greater robustness to respiratory motion [19] than standard parallel imaging techniques. Our C-SENSE protocol’s ability to assess biventricular haemodynamics and great vessel flow, which could be used to quantify valvular flow/regurgitant flow, in response to incremental exercise could theoretically provide a comprehensive assessment in valvular and congenital heart disease. Further research in these patient cohorts is required. In asymptomatic significant valve disease, ventricular dilatation/dysfunction or an abnormal exercise response can guide the decision to advise intervention [1, 34]. Given CMR is the reference standard for biventricular assessment and CMR derived aortic and mitral regurgitation quantification boasts superior prognostic value to transthoracic echocardiography [35–37], the additional assessment during exercise may hypothetically provide further prognostic information. Additionally, in-scanner MR-CPET is feasible [38] and our protocol could be performed in combination, theoretically creating a single comprehensive investigation. C-SENSE acceleration may benefit other Ex-CMR applications. For example, free breathing first pass perfusion using compressed sensing at rest [39] and supine exercise stress perfusion CMR are both feasible [40], therefore C-SENSE accelerated Ex-CMR stress perfusion may also be feasible. Our technique requires further research to demonstrate feasibility in patient populations, assess if additional prognostic information is provided above a resting CMR scan and whether C-SENSE can be used for other Ex-CMR applications.

### Study limitations

The study sample size is small and in healthy volunteers with a healthy mean BMI (23.9 ± 2.3 kg/m^2^) and a mean age (35 ± 9 years) younger than patients typically referred for exercise cardiac imaging. Supine Ex-CMR is feasible in older patients [41–44] and obese patients [45] but may be tolerated less well than by our study population, potentially resulting in more respiratory and motion artefacts. Thus our technique requires further evaluation in patients with cardiovascular disease. Derived volumes and flow from biventricular cine images and phase contrast images respectively were not compared directly with the reference standard of the direct Fick method, however as we have demonstrated, the biventricular cine and corresponding phase contrast flow stroke volumes correlated very strongly, demonstrating the internal validity of our technique. Additionally, our results follow prior supine Ex-CMR studies, as demonstrated in a recent meta-analysis [31], demonstrating rising stroke volumes with increasing exercise driven by a fall in LVESV but minimal change in LVEDV. Inter-scan reproducibility was not assessed with this study, but has been demonstrated in our institution previously in an Ex-CMR study assessing biventricular volumes using a similar retrospectively gated, respiratory navigated short axis cine sequence [42]. As expected, and demonstrated in prior Ex-CMR studies [16, 31, 46, 47], image quality decreases with increasing exercise intensity, however our study still demonstrated good intra- and inter-observer reproducibility during moderate intensity exercise. ECG interference was encountered in one patient, early in the study, such that miss-triggering occurred at moderate exercise intensity. This made analysis technically unfeasible and so the subject was excluded from the study. Subsequent subjects had pulse oximetry attached as a backup cardiac gating technique should ECG interference occur, however this was not required.

## Conclusion

Assessment of biventricular function, aortic and pulmonary flows during continuous exercise is feasible during exercise to moderate intensity using a free-breathing C-SENSE accelerated protocol. The ability to use commercially available analysis software with this vendor provided technique increases the potential clinical utility of Ex-CMR. The developed protocol allows the direct quantification of flow across the aortic and pulmonary valves and indirect quantification of mitral and tricuspid regurgitation during exercise. Further evaluation is needed in patients with cardiovascular disease to assess the value and reproducibility in a clinical setting.

## Abbreviations

BPM: Beats per minute
CAD: Coronary artery disease
CMR: Cardiovascular magnetic resonance
CPET: Cardiopulmonary exercise testing
CS3/CS6: Compressed SENSE encoding accelerated by a factor of 3/6
ECG: Electrocardiogram
EDV: End-diastolic volume
EF: Ejection fraction
ESV: End systolic volume
Ex-CMR: Exercise cardiovascular magnetic resonance
HR: Heart rate
HR_max_: Maximal heart rate
HRR: Heart rate reserve
i: Indexed to body surface area
LV: Left ventricle
LVEF: Left ventricular ejection fraction
LVOT: Left ventricular outflow tract
LVSV: Left Ventricular Stroke volume
MPS-SPECT: Myocardial perfusion scintigraphy by single photon emission computed tomography
MR: Mitral regurgitation
MR-CPET: Magnetic resonance cardiopulmonary exercise testing
PCMR: Phase contrast magnetic resonance
RVEF: Right ventricular ejection fraction
RVOT: Right ventricular outflow tract
RVSV: Right ventricular stroke volume
SENSE: Sensitivity encoding
SV: Stroke volume
THR: Target heart rate
